# Taxonomy, Diversity and Cultivation of the Oudemansielloid/Xeruloid Taxa *Hymenopellis, Mucidula, Oudemansiella,* and *Xerula* with Respect to Their Bioactivities: A Review

**DOI:** 10.3390/jof7010051

**Published:** 2021-01-13

**Authors:** Allen Grace Niego, Olivier Raspé, Naritsada Thongklang, Rawiwan Charoensup, Saisamorn Lumyong, Marc Stadler, Kevin D. Hyde

**Affiliations:** 1 Center of Excellence in Fungal Research, Mae Fah Luang University, Chiang Rai 57100, Thailand; agniego27@gmail.com (A.G.N.); ojmraspe@gmail.com (O.R.); fah_naritsada@hotmail.com (N.T.); 2 School of Science, Mae Fah Luang University, Chiang Rai 57100, Thailand; 3 Iloilo Science and Technology University, La Paz, Iloilo 5000, Philippines; 4 School of Integrative Medicine, Mae Fah Luang University, Chiang Rai 57100, Thailand; rawiwan.cha@mfu.ac.th; 5 Medicinal Plants Innovation Center, Mae Fah Luang University, Chiang Rai 57100, Thailand; 6 Department of Biology, Faculty of Science, Chiang Mai University, Chiang Mai 50200, Thailand; saisamorn.l@cmu.ac.th; 7 Research Center of Microbial Diversity and Sustainable Utilization, Chiang Mai University, Chiang Mai 50200, Thailand; 8 Academy of Science, The Royal Society of Thailand, Bangkok 10300, Thailand; 9 Department Microbial Drugs, Helmholtz Centre for Infection Research, 38124 Braunschweig, Germany; 10 German Centre for Infection Research (DZIF), Partner Site Hannover-Braunschweig, 38124 Braunschweig, Germany; 11 Institute of Plant Health, Zhongkai University of Agriculture and Engineering, Guangzhou 510408, China

**Keywords:** Basidiomycota, bioactive compounds, cultivation, diversity, taxonomy

## Abstract

The oudemansielloid/xeruloid taxa *Hymenopellis, Mucidula, Oudemansiella,* and *Xerula* are genera of Basidiomycota that constitute an important resource of bioactive compounds. Numerous studies have shown antimicrobial, anti-oxidative, anti-cancer, anti-inflammatory and other bioactivities of their extracts. The bioactive principles can be divided into two major groups: (a) hydrophilic polysaccharides with relatively high molecular weights and (b) low molecular medium polar secondary metabolites, such as the antifungal strobilurins. In this review, we summarize the state of the art on biodiversity, cultivation of the fungi and bioactivities of their secondary metabolites and discuss future applications. Although the strobilurins are well-documented, with commercial applications as agrochemical fungicides, there are also other known compounds from this group that have not yet been well-studied. Polysaccharides, dihydro-citrinone phenol A acid, scalusamides, and acetylenic lactones such as xerulin, also have potential applications in the nutraceutical, pharmaceutical and medicinal market and should be further explored. Further studies are recommended to isolate high quality bioactive compounds and fully understand their modes of action. Given that only few species of oudemansielloid/xeruloid mushrooms have been explored for their production of secondary metabolites, these taxa represent unexplored sources of potentially useful and novel bioactive metabolites.

## 1. Introduction

Basidiomycota, especially mushrooms, have been explored for thousands of years not only for their nutritional value but also as therapeutic agents [1,2]. Mushrooms are well-studied for their bioactivities such as anticancer, anti-diabetics, anti-hypertensive, antimicrobial, anti-inflammatory, anti-oxidant, immunomodulatory and cholesterol-lowering properties [2,3,4]. The discovery of mushroom metabolites pleuromutlin, illudin and strobilurins lead to the exploration of Basidiomycota as natural product-based candidates for drugs and agrochemicals [5,6,7]. On the other hand, medicinal mushrooms have long been used to treat diseases as traditional folk medicines in Asia [3]. Mushroom metabolites also have great potential to be developed as food supplements and additives for pharmaceutical and medicinal applications [5]. Producer organisms for commercial products and development candidates for such applications are mostly derived from genera such as *Agaricus* and *Ganoderma*, but include *Oudemansiella* [2,5,8,9] Recently, such studies have been increasingly relying on bioinformatics, genomics and transcriptomics [5,8].

The taxonomy of the oudemansielloid/xeruloid (OX) group, which comprises saprotrophic mushrooms that are widespread in all forested areas of the world, is quite complex and their generic classification has been re-arranged several times over the past decades. As the work of applied researchers such as chemists did not always keep pace with the taxonomy, there are a lot of synonyms in the literature that refer to certain species under different generic names. We here follow the concept derived from the major taxonomic study by Petersen and Hughes [10]. This work was based on comprehensive morphological studies including the reexamination of many types of materials and a concurrent molecular phylogeny based on rDNA data. The authors erected four new genera (*Hymenopellis*, *Paraxerula*, *Ponticulomyces*, *Protoxerula*) and reconfiguration of other genera such as *Dactylosporina*, *Mucidula*, *Oudemansiella* and *Xerula.* This concept has also been accepted in recent general overviews on the taxonomy of the Fungi and the Basidiomycota in particular [11]. 

Many studies have documented the bioactive compounds produced by these genera, which are dominated by strobilurins. These compounds, for which some other trivial names (oudemansins and mucidin) have been used in earlier publications, are all β-methoxy-acrylates with a similar carbon skeleton [8]. These compounds are known to have antifungal activity which are produced by mushrooms to eliminate competition from other fungi [12,13]. *Oudemansiella canarii* also produces oudemansin A with antimicrobial properties [14]. There are also numerous studies on the polysaccharides from these genera with focus on health-promoting activities. Due to the increased awareness in the pharmaceutical and nutritional values of mushrooms, there is an amplified demand from consumers for other varieties of mushrooms, thus leading to the exploration of wild mushrooms for utilization [15]. There are over 30,000 species of Basidiomycota in the world [5], of which only a small percentage (5%) has been investigated [13].

This paper aims to explore the OX group of mushrooms as sources of bioactive compounds. We highlight the importance of this group by gathering information on the bioactivities and compounds produced from the earliest records up to the present. Furthermore, we discuss their diversity, distribution, taxonomy and different methods for their cultivation. 

## 2. Taxonomic Aspects of Oudemansielloid/Xeruloid Genera

*Hymenopellis*, *Mucidula, Oudemansiella* and *Xerula* are Physalacriaceae genera that share a complicated taxonomical history (Table 1). These species complexes have been dealt with by different mycologists in order to clarify their classification during the past 140 years and some important papers are mentioned below. *Oudemansiella* was initially proposed as *Oudemansia* in order to accommodate a single species, *Agaricus platensis* [16]. Spegazzini [17] then changed the name to *Oudemansiella*. Moser [18] merged the genera *Xerula* and *Mucidula* under *Oudemansiella.* This arrangement was supported and adopted by Singer [19,20,21], but *Xerula* was regarded as a subgenus only within *Oudemansiella*. Clemencon [22] also treated *Xerula* as one of the five subgenera in *Oudemansiella*. Dörfelt [23], however, retained *Oudemansiella* and *Xerula* as two independent genera and this was adopted by other researchers [24,25,26,27,28,29,30,31,32,33,34]. Pegler and Young [35] divided *Oudemansiella* into 5 sections under the two subgenera *Oudemansiella* and *Xerula.* Other mycologists such as Rexer and Kost [36,37], Yang and Zang [38], Yang [39] and Mizuta [40] later adopted this new arrangement.

Yang et al. [41] proposed a taxonomic classification for the genus *Oudemansiella* s.s., which was divided into four sections, i.e*., Oudemansiella*, *Mucidula*, *Dactylosporina* and *Radicatae*. Section *Oudemansiella* comprised tropical to south temperate species, e.g., *O. platensis, O. australis, O. canarii* and *O. crassifolia*. The distinguishing characteristics of this group was the ixotrichoderm pileipellis composed of filamentous hyphae often intermixed with chains of inflated cells. The section *Mucidula* on the other hand was characterised by an ixohymeniderm-trichoderm pileipellis composed of more or less clavate terminal cells and encompassed north temperate and subtropical taxa (e.g., *O. mucida*, *O. venosolamellata* and *O. submucida*). Sections *Oudemansiella* and *Mucidula* shared similar habitats, growing on exposed rotten wood. Their basidiomata were with or without a (rudimentary) annulus on the stipe. Section *Dactylosporina* accommodated species from South and Central America with basidiospores that had finger-like ornamentation. Section *Radicatae*, represented by *O. radicata* and its allies, was the largest section and included the remaining species of the genus in its restricted sense.

**Figure 1:**
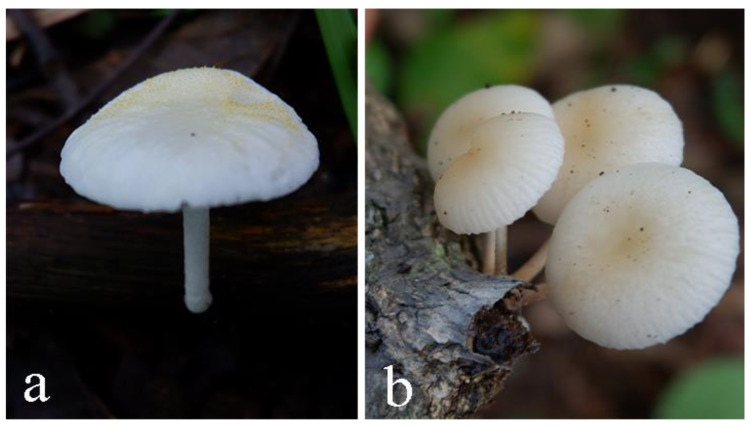
Basidiomata of *Oudemansiella* collected from the wild in Thailand. (**a**,**b**). *Oudemansiella* spp. (HT19-0047, HT19-0050). Photos by A.G. Niego.

So far the most thorough revision of the OX complex was provided by Petersen and Hughes [10], and it is still widely accepted today [11,12]. Based on taxonomic and phylogenetic analyses, 68 new taxa/or new combinations were proposed. The new arrangement included introduction of new genera (*Hymenopellis, Paraxerula, Ponticulomyces* and *Protoxerula*) and reconfiguration of *Dactylosporina, Mucidula, Oudemansiella* and *Xerula*. For instance, *Oudemansiella* and *Mucidula* grow directly on wood without developing pseudo-rrhizae. Macroscopically, the basidiomata of *Oudemansiella* differ from those of *Mucidula* by lacking a persistent annulus on the stipe and the former genus can only be found in tropical areas. There are 142 records of names in Index Fungorum [41], with 39 currently accepted species for *Oudemansiella*. *Mucidula,* on the other-hand, was introduced by Patouillard [42] and 14 records of the genus are presently listed, of which only *Mucidula brunneomarginata* and *Mucidula mucida* are currently accepted [10]. Figure 1 shows some specimens of *Oudemansiella* collected from Thailand.

**Figure 2:**
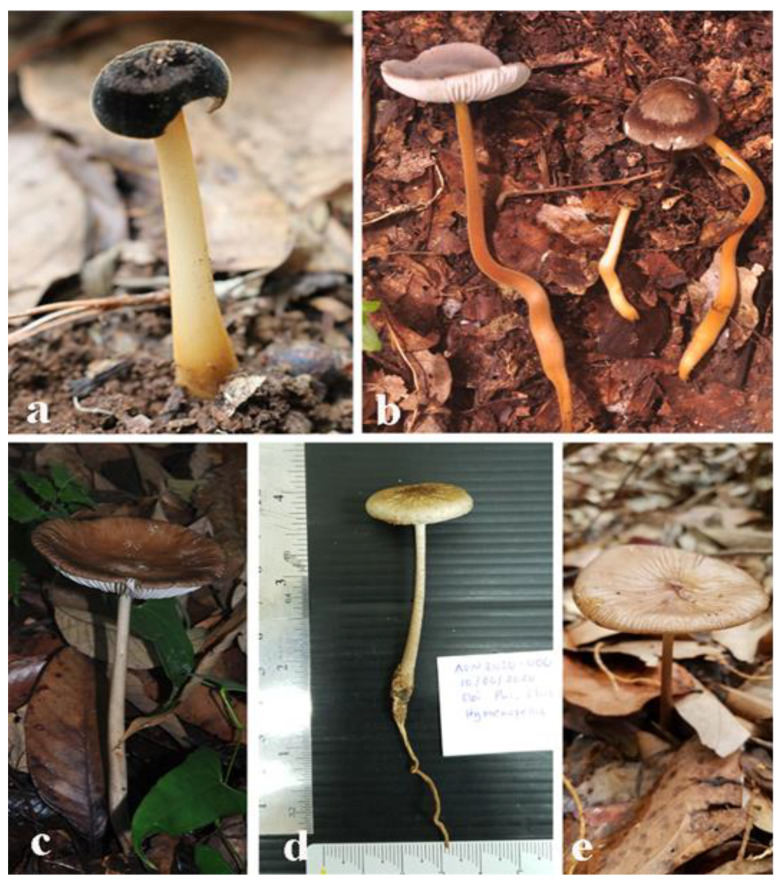
Some basidiomata collected from the wild in Thailand. (**a**). *Xerula* sp. (immature basidioma) (**b**). *Xerula sinopudens* (**c**–**e**). *Hymenopellis* sp. Photos by A.G. Niego.

The basidiomes of the other, related genera have pseudorhizae extending below ground and connected to subterraneous wood or tree roots. *Xerula* was described by Maire [45], and currently there are 96 records in Index Fungorum, including the synonyms, of which only 11 species accepted. This genus differs from *Paraxerula* by having thick-walled setae on the pileus [10]. Moreover, basidiomes of *Hymenopellis* species have a moist to glutinous pileus, in contrast to *Protoxerula*, which has a green, sticky pileus and is restricted to Australia. The type species of *Hymenopellis* is *H. radicata* described in 1786 under the name *Agaricus radicatus* [48]. There are 58 records with 42 species for *Hymenopellis* in Index Fungorum. Figure 2 shows some specimens of *Xerula* and *Hymenopellis* collected from Thailand.

## 3. Geographical Distribution and Diversity of the Genera

### 3.1. Hymenopellis

**Figure 3:**
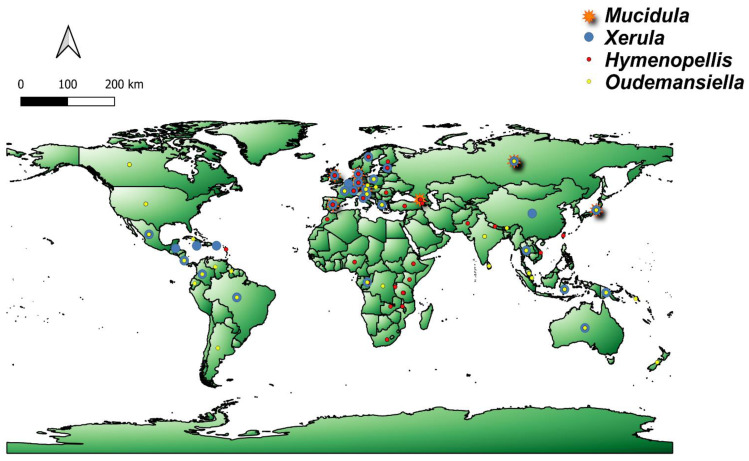
Geographical distribution of OX genera showing their concentration in some continents.

*Hymenopellis* species are widely distributed in eastern and north America [49] (Figure 3). Although the distribution is well documented in these areas, they can also be found in other continents. Many species of *Hymenopellis* were first documented in Asia. For instance, *H. amygdaliformis* and *H. velata* were first found in China [10,38], *H. aureocystidiata, H. japonica, H. orientalis* and *H. vinocontusa* in Japan, and *H. endochorda* in Sri Lanka [10]. *Hymenopellis chiangmaiae* was first recorded in Thailand but was later synonymized under *H. raphanipes* by Petersen and Hughes [10]. Several species of the genus have also been discovered in Australia. These are *H. eradicata, H. gigaspora, H. mundroola, H. superbiens, H. trichofera* and *H. variabilis*. Other species are distributed almost worldwide, such as *H. radicata.* This fungus occurs in Europe and North America and can also be found in northern Africa, in extreme western Asia and Asia minor [10,49,50]. *Hymenopellis raphanipes* was first described from India [51] and has also been reported from Australia, China, India, Japan and Thailand [10,26,35,38]. Basidiomata of *Hymenopellis* can grow solitary or gregarious on dead or buried hardwoods, and occasionally on exposed, well-decayed wood. They can appear as growing from the ground because of their long deep tap-root like pseudorhiza attached to the decayed wood underground [10].

### 3.2. Mucidula

*Mucidula mucida* var*. mucida*, the “porcelain mushroom”, is commonly found and widespread in Europe including western Russia and typically grows on *Fagus* [10] (Figure 3). Other varieties, *Mucidula mucida* var*. asiatica* and var*. venosolamellata*, are distributed in Asia. In Japan, *M. mucida* var*. asiatica* has been collected from dead trunks and branches of several broad-leaved tree species, while *M. mucidula* var*. venosolamellata* usually grows in dead trunks and branches of *Fagus crenata* [52]. The second species in the genus, *Mucidula brunneomarginata*, is commonly found on rotting hardwood logs. It was first recorded in Russia and has also been documented in Japan [10,53].

### 3.3. Oudemansiella

*Oudemansiella* is widely distributed throughout tropical and temperate regions (Figure 3) and its basidiomata grow on rotting wood [10,11]. For instance*, Oudemansiella canarii* can be found in Asia, Africa and Central America [14,21,43,54]. *Oudemansiella platensis* var*. orinocensis* can also be found in tropical and subtropical regions [55]. Many species of *Oudemansiella* were first recorded in Asia, as exemplified by *O. alphitophylla* (as *Agaricus alphitophyllus*), *O. latilamellata*, and *O. rhodophylla* [40,56], which were all first recorded in Japan. On the other hand, *Oudemansiella bii*, *O. fanjingshanensis* and *O. yunnanensis* were first recorded in China. Others, like *O. crassifolia* and *O. submucida* were first recorded in Malaysia, and have also been recorded in Thailand [32,54]. In some cases, it is not possible to say for sure whether they belong to other genera described here because the descriptions did not rely on the concept by Peterson and Hughes [10]. For instance, *O. submicida* is probably better placed in *Mucidula* as it closely resembles *M. mucida*. This shows that a lot of work remains to be done to harmonize the taxonomy of the OX complex at a global level. Some species of *Oudemansiella* such as *O. exannulata*, *O. gloriosa*, *O. reticulata* and *O. turbinispora* appear to be endemic to Australia [10,57]. Historically, Europe is the best studied of all the continents in terms of the number of publications of this genus; however, in terms of the number of species, Asia seems to be more diverse [10], and Africa, as well as South and Central America seem to be understudied.

### 3.4. Xerula

The type species of the genus, *Xerula pudens* (often treated in the literature under its synonyms, *Xerula* or *Oudemansiella longipes*) has been reported first in Europe (cf. Figure 3). This species is connected to *Quercus*, thus its distribution could theoretically cover the whole continent [50,58]. It has also been reported in Thailand [59], but without details on the morphology, hence this record is highly dubious because *Quercus* does not actually occur in that country. The first recorded Asian species are *Xerula sinopudens* in Japan and *Xerula strigosa* in China [26,41]. Other species were documented in different countries, such as *X. australis* (Australia), *X. fraudulenta* (France), *X. oronga* (D.R. Congo), *X. renati* (Switzerland, as *Oudemansiella renati*) and *X. setulosa* (Jamaica, as *Gymnopus setulosus*) [28,60,61,62,63]. The latter species was also documented in Brazil and Belize [27,64]. Generally, the species are similar to *Hymenopellis* in being saprobic, and their basidiomata are attached to rotten wood, which is often buried deep beneath leaf litter or soil [10].

## 4. Cultivation of Important Species with Bioactivities

Generally, mushrooms are cultivated for food because of their good taste and high nutritional value. In Japan and China, mushrooms are traditionally consumed because of their medicinal and tonic properties [65]. Mushroom cultivation is an important part of sustainable agriculture and forestry. Cultivation is necessary to ensure a stable mushroom source especially for potential sources of bioactive compounds. It can also help small farming systems by recycling agricultural wastes and returning them to soil as fertilizer [66]. The most widely known cultivated mushrooms are *Agaricus bisporus* and *Volvariella volvacea*, which represent almost 38% and 16% of total mushroom production in the world [67]. In any case, the empirical optimization of culture conditions is necessary to assure that the respective biotechnological production processes is competitive and commercially viable before such products can be introduced into the market.

**Figure 4:**
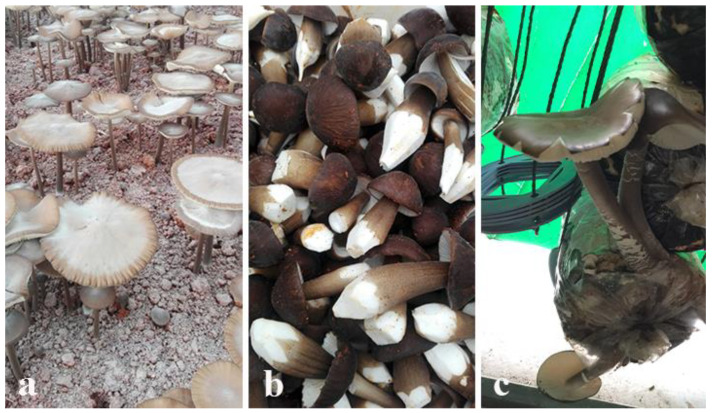
Basidiomata of *Hymenopellis raphanipes* cultivated in China and in the Mae Fah Luang (MFU) laboratory, Thailand. (**a**). Mature basidiomata, (**b**). Young basidiomata, (**c**). Basidomata from bags. Photos from Yu Wei and A.G. Niego.

OX mushrooms are not generally commercially cultivated as food source as the fruiting bodies are rubbery and do not have good taste. Only in some Asian countries, such as China, are these mushrooms being commercially cultivating at larger scale, presumably mostly for medicinal purposes. Aside from *Hymenopellis radicata,* the cultivation of no other edible member of the OX group has been documented in detail (Figure 4). However, several species that are used as medicinal mushrooms have been successfully grown on different substrates at laboratory scale with high biological efficiency (Table 2). The later term refers to the percentage of ratio of fresh mushroom weight vs. the dry weight of the respective substrate [68].

### 4.1. Cultivation of Hymenopellis

Kim et al. [74] were able to establish the optimal culture conditions for mycelial growth of *H. radicata* at 25 °C and pH 6.0. This species was successfully grown on sawdust, with biological efficiency of 100% [70]. The addition of 10% rice bran to oak sawdust stimulated mycelial growth since it may contain ingredients favourable for mycelial growth for *H. radicata* [69]. 

*Hymenopellis raphanipes* is commercially cultivated in China by the local name “Heipijizong” or “Black Termite Mushroom”. It was previous misidentified as *H. furfuracea, H. radicata, Termitomyces fuliginosus* or *T. badius*. Hao et al. [75] correctly identified this mushroom by using morphologic and phylogenetic (ITS and nrLSU) analyses. The results clarified the phylogenetic position and taxonomy of “Heipijizong” as *H. raphanipes.* To increase production for large scale cultivation, the use of liquid culture fermentation and optimization of culture conditions of fermentation technology was studied by Ning et al. [76].

The best medium for liquid culture fermentation was glucose 20.0 g + sorghum powder 4.0 g + K_2_HPO_4_ 3.0 g + MgSO_4_ 1.0 g + vitamin B1 2 tablets + distilled water 1000.0 mL, pH 6.5. The 12% of inoculum was grown in 2 L liquid having an optimum temperature of 25 °C, stirring speed 90 r/min, culture time 100 h, tank pressure 0.3 MPa and ventilation volume 0.9 m^3^/h. By using optimum conditions, the mycelium of the *O. raphanipes* cultivated in the liquid medium had a fast growth rate, filling the bag in 29 days, with an average yield of 360.0 g per bag. The biological conversion rate reached 78%. Figure 4 shows photographs of some strains taken in China.

### 4.2. Cultivation of Mucidula

*Mucidula mucida* can be grown in Potato Dextrose Agar at 25 °C [75]. It can also successfully grow in nutrient media for mycelial growth. Musilek et al. [77] grew this species on glucose-corn-steep media containing 30 or 50 g glucose, 15 g corn-steep (~50 dry weight), 1.5 g MgSO_4_.7H_2_O per liter of water with the pH 5.5. This species was successfully cultivated in oak sawdust mixed with rice bran (20–30%) in the bottle at 25 °C, incubated in the dark [71]. The mycelia then colonized the media from the top to bottom. The bottles were exposed under 12 h of light (350 lux) and dark having a relative humidity of 95% at 17 °C. The primordia were observed after 7 days of incubation. They then developed into mature fruiting bodies after 7 days [71].

### 4.3. Cultivation of Oudemansiella

The most common substrate used in the cultivation of *Oudemansiella* species is sawdust. All cultivable species of *Oudemansiella* can be grown in this substrate [72,73,78,79]. Recently, however, other substrates have been used (Table 2). 

Among the species of *Oudemansiella*, *O. canarii* is the most commonly cultivated. It can be grown in different biomass, since this species is able to colonize several kinds of plant. Silveira Ruegger et al. [72] cultivated *O. canarii* strain CCB179 in polypropylene bags in two different substrates (200 g), sugar-cane bagasse and eucalyptus sawdust incorporated with wheat bran (50 g). The composts were sterilized at 121 °C for an hour and were later inoculated with 3 g of spawn. The bags were incubated at 25 °C until the basidiomata primordia formed. Mushroom growing in sugar-cane bagasse resulted in higher biological efficiency (55.66%) as compared with eucalyptus sawdust supplemented with wheat bran (19.51%). Lignocellulosic wastes such as cottonseed hull and corn-comb can also be used as substrates in cultivating *O. canarii*. Xu et al. [73] used these lignocellulosic wastes as base substrates for the cultivation of *O. canarii*. The addition of wheat bran and lime in the substrates provide nitrogen and adjusted the pH of the substrates. 1000 g of substrate were prepared in each bag with water content adjusted to 65% (*w*/*w*). The substrates were then inoculated with spawn at 2% (*w*/*w*). The bags were then incubated at 25 °C and 70% relative humidity (RH) in the dark room. Among the substrates cotton-hull (80%) resulted in highest biological efficiency (113.64) and essential amino acid contents. The combination of cottonseed hull (80%) supplemented with 8% wheat bran and 2% lime can give a high yield of basidiomata and should be extended in future use [73]. 

*Oudemansiella submucida* has also been domesticated and successfully cultivated. A wild strain from Hunan Province has been domesticated by Li et al. [79]. The optimal conditions for primordial growth are 23 °C and 75–95% relative humidity. After 40–50 days following substrate inoculation the primordia appeared. The biological efficiency of 96.1% was obtained from the first flush after 50–55 days.

### 4.4. Cultivation of Xerula

The artificial cultivation method of *Xerula pudens* (CN104396561A) was patented by Houjiang et al. [80] The culture medium was prepared by degumming, degreasing and curing of saw-dusts from pine and rubber trees. The medium comprised 85 to 92 parts of any sawdust or sawdust mixtures. Additives such as 5 to 10 parts of broad bean husks and 3 to 5 parts of bean pulp were incorporated into the medium. The water content was about 50–70%. The culture medium was placed in plastic bags and sterilized. After cooling, they were inoculated with the mycelial culture under aseptic conditions. The bags were covered with vermiculite for fructification. This method is low cost with high yield of fruiting bodies which could be extensively applied to large-scale cultivation and production of *X. pudens.*

## 5. Bioactivities and Mode of Action

Basidiomycota have long been recognized as sources of interesting secondary metabolites; however, because of the slow mycelial growth and diverse nutritional requirements they were often been neglected as a source of important bioactive compounds [5,81]. Recently, due to the progress in -Omics technology, such as improved fermentation technologies and the development of sophisticated chemical analysis methods for secondary metabolites, the chances of developing new products from Basidiomycota have increased considerably [8,82,83,84].

Aside from low molecular metabolites, they contain beta-glucans and other oligomers that constitute the active ingredients of medicinal mushrooms [3,4,85,86,87]. The Physalacriaceae are known to produce manifold antibiotics from mycelial cultures [8,88], but can also produce polysaccharides with anticancer, antihypertensive, anti-inflammatory and hemagglutination activities [89,90,91,92]. Many studies have documented the bioactivities of these mushrooms; however, most were inconclusive since they did not go further in identifying the active principles using chromatography and spectral techniques, such as mass spectrometry and nuclear magnetic resonance (NMR) spectroscopy. Table 3 lists some metabolites isolated from OX genera and their bioactivities.

The challenge of finding antimicrobial agents has become evident due to the increasing resistance of pathogenic microorganisms to present-day drugs [2]. The exploration of bioactive compounds especially from natural sources such as plants, bacteria and fungi is needed to develop less toxic and more potent antibiotics [93]. Recently, mushrooms have been subjected to screening for bioactive compounds and many studies have revealed their antimicrobial activities [2,94]. The Agaricales were explored for their antimicrobial capacity. The antimicrobial properties of mushrooms have potential in the defense against several diseases [95]. *Oudemansiella canarii* has been well studied for its significant antimicrobial activities against *Candida albicans, C. glabrata*, *C. krusei*, *C. tropicalis* and *C. sphaerospermum* [13,14,96]. The extract of *Mucidula mucida* (as *O. mucida*) was shown to have antibacterial activity against the Gram positive bacterial pathogen, Staphylococcus aureus [95], but this cannot be explained by the presence of strobilurins, which are selective antifungal agents. Therefore, oudemansielloid species could turn out to be a source of novel potent compounds with antimicrobial properties, once they have been studied more thoroughly.

**Figure 5:**
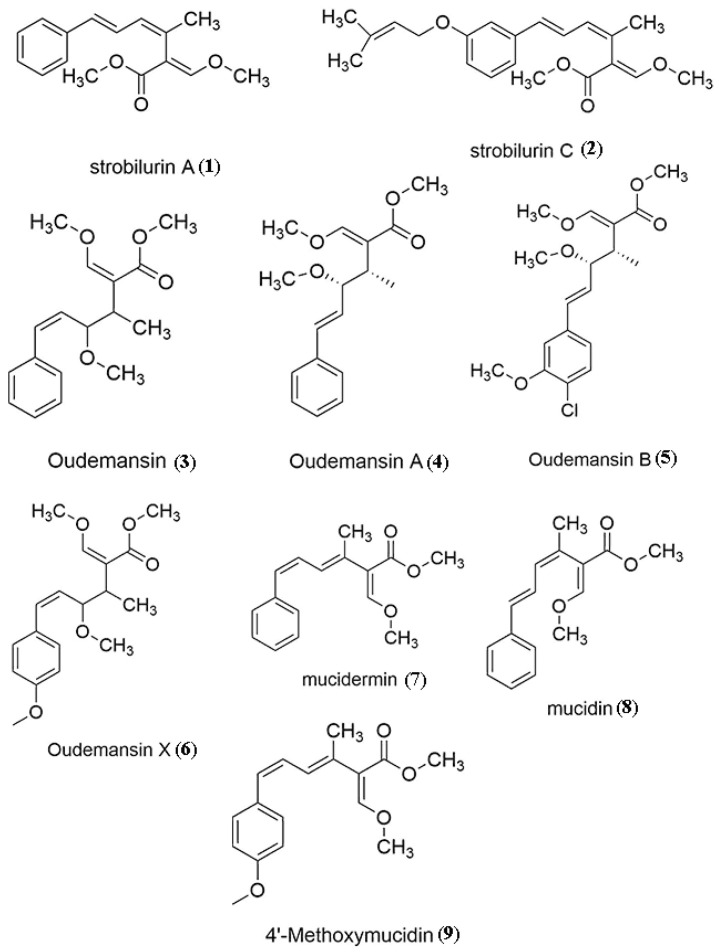
Chemical structures of bioactive compounds isolated from OX genera with antimicrobial/antifungal activities.

Some compounds from OX taxa are already well studied. The strobilurins, first reported by Anke et al. [97] from fermentations of *Strobilurus tenacellus*, were later also isolated from numerous other Basidiomycota. The species *H. radicata* (as *O. radicata* and *M. mucida* (as *O. mucida*) were also able to produce this compound and its derivatives (strobilurins A (4), B (5) and X (6) [14,98,99,100] (Figure 5). The trivial names of these natural fungicides are based on the order of their discovery (Table 3, Figure 5). They are potent inhibitors of respiration owing to their ability to inhibit electron transfer between mitochondrial cytochrome b and cytochrome c1 through binding at the ubiquinol-oxidation centre [101,102]. This development opened the door to new synthetic fungicides. The synthetic analogues based on Quantitative Structural Activity Relationships (QSAR) of the structures of the natural strobilurins are more effective and stable [103].

Oudemansins and strobilurins have been reported from a variety of Basidiomycota, which are widely distributed all over the world in tropical and temperate climates, but the most frequently reported producers are the genera of the Physalacriaceae treated here and the related genera *Strobilurus* and *Mycena* [114]. Mycelial cultures of *M. mucida* produce oudemansin A, which is closely related to strobilurin A [104], and these compounds show high antifungal activity at very low concentrations [77,115]. Oudemansin also inhibited the growth of Ehrlich ascites carcinoma in rats, but at rather weak concentrations [105]. Rosa et al. [14] later found the compound in *O. canarii*. An extract of cultures of the latter species showed antifungal effects but inhibited the growth of UACC-62 cells by 47% and the enzyme trypanothione reductase (TryR), thus indicating anti-tumor activity. *Hymenopellis radicata* is also known to produce oudemansins [14,100]. Mucidin and strobilurin A were found to be identical [84,102]. Mucidin was discovered in the 1960s from the submerged culture of *M. mucida* as an antifungal agent and its structure was described and established in 1979 [98]. Šubík et al. [104] noted that mucidin could inhibit the growth and germination of the conidia of *Aspergillus niger*. It also completely prevented the growth of yeasts in glycerol and ethanol and inhibited the growth of wild-type yeasts including anaerobes. Mucidin repressed the oxidation of glucose and ethanol under aerobic condition; however, in an anaerobic environment, the metabolism of glucose was not affected. In the presence of glucose, mucidin was able to reduce cytochrome b and completely oxidized cytochrome a and c by inhibiting mitochondrial electron transport, thus contributing to its antifungal activity. Specifically, it inhibits electron-transfer reactions in the cytochrome bc1 complex of the mitochondrial respiratory chain [102]. 

A water-soluble polysaccharide (ORWP) from *Hymenopellis radicata* also inhibited the growth mould *P. digitatum* by disrupting the hyphal membrane, leading to leakage of intracellular materials, and impaired cellular metabolism [116]. There are other promising compounds identified with antimicrobial activities which do not belong to the group of strobilurins. One is scalusamide A, an antifungal agent and its derivatives (B,C) isolated from *Xerula* sp. BCC56836 [113].

Many studies have been conducted on the anti-oxidant and anti-cancer properties of other oudemansielloid species, which cannot be explained by the presence of strobilurins. As early as 1987, *Mucidula mucida* extracts demonstrated inhibitory effect on sarcoma 180 and Erhrlich carcinoma of mice [105]. *Oudemansiella canarii* has moderate anticancer and anti-oxidant properties [96,117]. The 2,2-diphenyl-1-picrylhydrazyl (DPPH) radical scavenging, total antioxidant activity and 2,2-azinobis (3-ethyl benzothiaoline-6-sulfonic acid) (ABTS) assays were used to determine anti-oxidation properties of the methanolic extracts of *O. canarii* [117]. HPLC analysis was also used to record and analyse phenolic fingerprints. Acharya et al. [117] were able to quantify the DPPH radical scavenging activity using EC_50_ at 0.912 µg/mL. The total antioxidant activity was 15.33 µg ascorbic acid equivalent/mg of extract. ABTS revealed 12.91 µm TE/mg of extract antioxidant activity. *Oudemansiella canarii* can therefore be a novel source of antioxidants with functional food and supplement applications. An ethyl acetate extract from *O. canarii* was active against enzyme TryR from *Trypanosoma cruzi*, three human cancer cell lines (MCF-7- breast, TK-10- renal and UACC-62-melanoma) and phytopathogenic fungus *Cladosporium sphaerospermum* [96]. However, in its activity against three cancer cell lines, it was noted that it exhibited a degree of selectivity against UACC-62, 2–3 times more active as compared with MCF-7 and TK-10. As an important note, the extract was inactive in lymphocyte proliferation assays, thus indicating that this compound has a low level of toxicity to normal human cells. It is therefore likely to be safe when used and developed in pharmaceutical applications [96].

**Figure 6:**
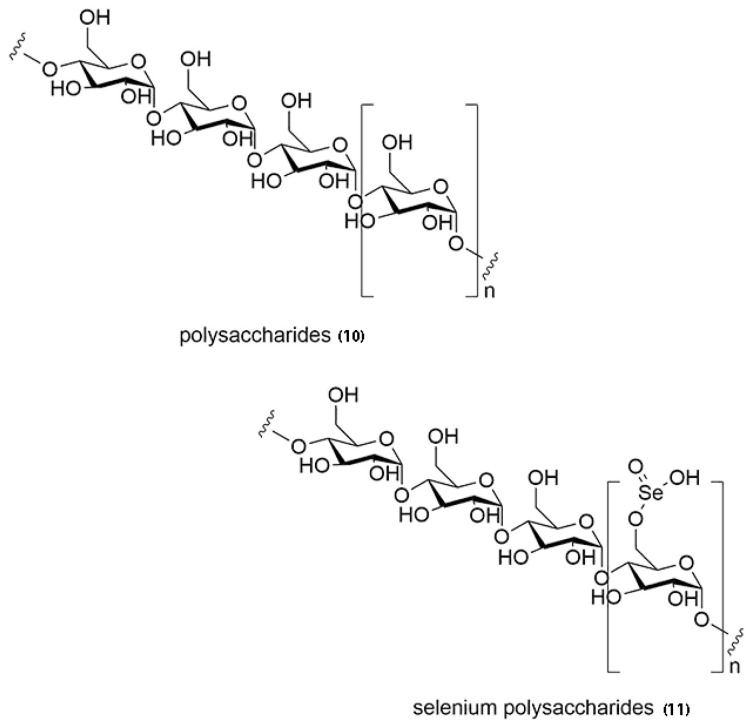
Chemical structures of polysaccharides isolated from OX genera with anti-oxidative properties.

Polysaccharides isolated from the oudemansielloid genera showed anti-oxidative properties [9,91,116,118]. Polysaccharides such as water-soluble polysaccharides (ORWP) and alkali-soluble polysaccharides (ORAP) from *Hymenopellis radicata* were tested for in vitro antioxidant and in vivo hepatoprotective activities [9,116]. The polysaccharides displayed anti-oxidative activity against CCl4-induced liver injury of mice, thus demonstrating the hepato-protective effect of these compounds [9]. Mycelia polysaccharides (MPS) and mycelia selenium polysaccharides (MSPS) (Figure 6) isolated from *H. radicata* also have antioxidative and lung-protective effects [92,118]. The MSPS derived from the fungus was able to relieve lung injury and prevent oxidative stress from lipopolysaccharide-induced lung injured mice, thus they can possibly be developed into functional foods and natural drugs in preventing lung injury [91]. These polysaccharides showed potential for relieving liver injury by monitoring the serum levels of hypersensitive C-reactive proteins, complement 3, and serum enzyme activities (aspartate aminotransferase, alanine aminotransferase, and alkaline phosphatase). They also enhanced antioxidant enzyme abilities (superoxide dismutase, glutathione peroxidase, catalase, and total antioxidant capacity). Lipid peroxidation (lipid peroxidation and malondialdehyde) also decreased. The polysaccharides were mainly composed of mannose, glucose and galactose as monosaccharide components [116].

The enzymatic- and acid- hydrolysed mycelia polysaccharides (En-MPS and Ac-MPS) from *Hymenopellis radicata* on lipopolysaccharide-induced acute lung injury (ALI) mice was tested for their antioxidative and pulmonary protective effects [118]. En-MPS has more antioxidative effect than Ac-MPS. Selenium polysaccharides were also produced by *H. radicata*. The hydrolysates (enzymatic-SPS) and acidic-SPS) were acquired by enzymolysis and acidolysis. The in vivo mice experiments showed that the enzymatic-SPS displayed higher antioxidant and protective effects against the lipo-poly-ssachardide-toxicities than selenium polysaccharides and acidic-SPS by increasing the antioxidant activities and reducing lipid peroxidation. Enzymatic-SPS also helps improve the inflammatory response which could aid in improving kidney and lung functions. This shows that the polysaccharides by *H. radicata* might be apt for functional foods. They can also be developed as natural drugs in preventing the endo-toxemia and its complications [118]. Figure 6 shows the chemical structures of polysaccharides and salinised polysaccharides.

The most commonly administrated drugs to reduce inflammation in the body are presently nonsteroidal anti-inflammatory drugs (NSAIDs). The negative effects of long-term use of these drugs, especially their significant side effects on the gastrointestinal tract, are well-known [119,120,121]. Therefore, much effort has been devoted to the search for novel compounds as alternative anti-inflammatory agents that would be natural and safe, without the harmful side effects of NSAIDs [122]. Mushrooms have been explored for their favourable therapeutic and health-promoting benefits, particularly in relation to diseases associated with inflammation [2]. Compounds with highly diversified chemical structures and anti-inflammatory activities have been isolated and purified from different types of mushrooms. Mushrooms, such as those from the oudemansielloid genera, are rich in anti-inflammatory components, such as polysaccharides, phenolic and indolic compounds [123]. The antioxidant activity of extracts is mostly coupled with anti-inflammatory effects. The enzymatic-mycelia polysaccharides and acid-hydrolysed mycelia polysaccharides from *Hymenopellis radicata*, for example, have anti-inflammatory effect aside from the antioxidative and pulmonary protective activities of the mycelial selenium-enriched polysaccharides and mycelial polysaccharides from other studies [92,116,118]. The anti-inflammatory and reno-protective effects of selenized mycelial polysaccharides from the same species have also been reported [124]. Further studies should be conducted to identify and elucidate the bioactive compounds from OX genera responsible for its anti-inflammatory properties.

Edible mushrooms have the ability to stimulate the immune system by exerting effects on cellular activities, producing secondary metabolites that boost the immune system, modulate humoral and cellular immunity, and potentiate antimutagenic and antitumorigenic activity, as well as rejuvenating the immune system destroyed by radiation and chemotherapy in cancer treatment, usually linked to β-glucans [125,126]. Specifically, β-glucan, a water-soluble polysaccharide, activates immune cells and proteins and macrophages, T cells, natural killer cells, and cytokines that attack tumor cells [127,128]. This potential of mushrooms, therefore, qualifies them as candidates for immunomodulation and immunotherapy in cancer and other disease treatments [128]. In addition, lectins from mushrooms have many biological activities, such as antiproliferative, antitumor, immunomodulatory, and HIV-1 reverse transcriptase inhibiting activities [3,129].

**Figure 7:**
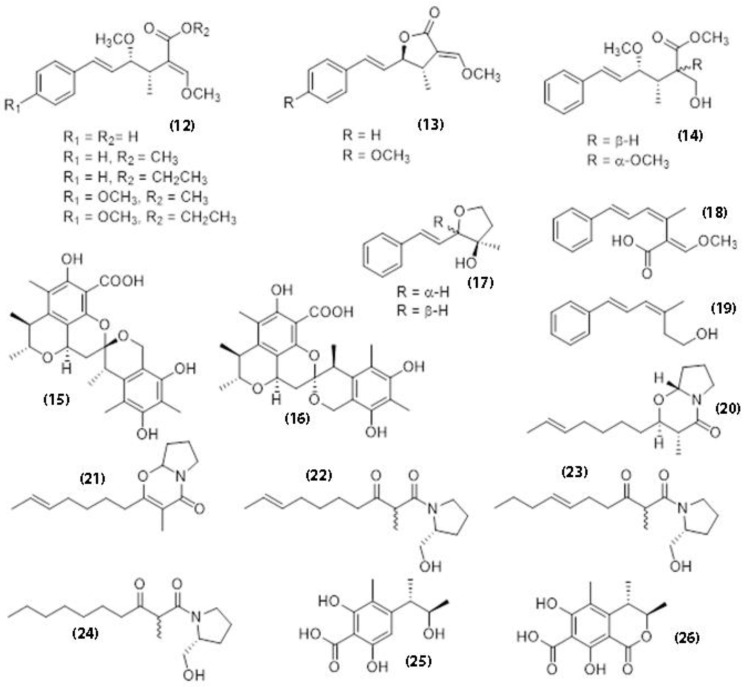
Chemical structures of compounds identified from *Xerula* sp. BCC56836 with antifungal and insecticidal properties.

Sadorn et al. [113] identified 12 (Figure 7) different compounds from a *Xerula* sp. (strain BCC56836) in Thailand. These were mostly known compounds, such as oudemansins, derivative of strobilurin, scalusamides A–C, phenol A acid and di-hydro-citrinone. The strain also produced compounds such 2-(5-heptenyl)-6,7,8,8a-tetrahydro-3-methyl-4H-pyrrolo [2,1-b][1,3]oxazin-4-one with insecticidal activity. Some oudemansins known for their antifungal activity also exhibit antimalarial activity against *Plasmodium falciparum* (IC_50_ 1.19–13.7 µM) and antifungal activity against *Alternaria brassicicola* and *Colletotrichum capsici* with MIC values ranging from 12.5−50 µg/mL. Aside from antifungal and antibacterial activities of 2-(E-hept-5-en-1-yl)-3-methyl-6,7,8,8a-tetrahydro-4H-pyrrolo[2,1-b][1,3]oxazin-4-one, the compound also has insecticidal activity against the four-instar *Oncopeltus fasciatus* (milkweed bug) and anti-phyto-pathogenicity against *Fusarium culmorum*, *Colletotrichum coccodes*, *Alternaria tenuis*, and *Penicillium italicum*. The compounds phenol A acid and di-hydro-citrinone have enzyme-inhibitory activity against cathepsin B with IC_50_ values of 20.4 ± 1.9 and 28.5 ± 1.7 µM, respectively. Most of the compounds isolated have low cytotoxicity in both the both cancerous and non-cancerous cells.

**Figure 8:**
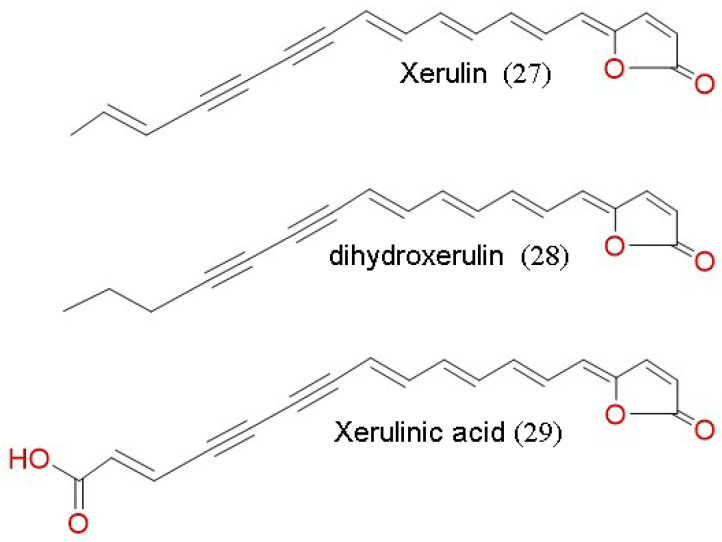
Chemical structures of other compounds act as inhibitors of cholesterol biosynthesis isolated from *Oudemansiella melanotricha.*

Xerulins (27) with their derivatives, di-hydro-xerulin (28) and xerulinic acid (29) (Figure 8) were isolated from *Oudemansiella melanotricha*. These compounds act as inhibitors of cholesterol biosynthesis. They strongly inhibit the incorporation of ^14^C acetate into cholesterol in HeLa cells [112]. Xerulin and di-hydro-xerulin inhibited the biosynthesis of cholesterol in HeLa S3 cells (ID_50_ = 1 µg/mL) without being cytotoxic [111,130]. Xerulinic acid, however, also inhibited biosynthesis but was found to be cytotoxic [111].

Oudenone from cultures of *Hymenopellis radicata* is an inhibitor of tyrosine hydrolase, an enzyme responsible for catalysing the conversion of the amino acid L-tyrosine to L-3,4-dihydroxyphenylalanine, thus it could have potential as antihypertensive agent [89,90,107].

Ingestible polysaccharides are the main components of mushrooms that play a prebiotic role by modulating the composition of gut microbiota [110]. Liu et al. [110] showed that polysaccharides from *Hymenopellis radicata* (as *Oudemansiella radicata*) were utilized by gut microbes to produce short-chain fatty acids during anaerobic fermentation of indigestible polysaccharides, therefore regulating the composition of gut microbiota. Hence the polysaccharides found in this mushroom cold be developed into a functional food that promotes intestinal health and prevents diseases.

## 6. Biosynthesis of Strobilurins and Total Synthesis of Xerulins

**Figure 9:**
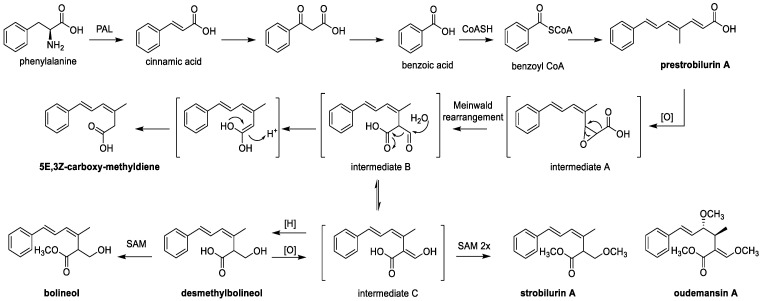
Biosynthesis of strobilurin A [85].

The bio-synthethic gene cluster for strobilurin was first identified from *Strobilurus* sp.; however, its detailed molecular biosynthesis remains cryptic. Nofiani et al. [84] reported the biosynthesis of strobilurin using *Aspergillus oryzae* by identifying the biosynthesis gene cluster, which encodes the highly reducing polyketide synthase. The synthesis is via a novel route initiated with benzoyl CoA molecules rather than the usual acetyl unit (Figure 9). The compound is formed by the degradation of phenylalanine via cinnamate [99]. As the core polyketide chain is formed, it undergoes a complex rearrangement to make the β-meth-oxy-acrylate toxophore. The antifungal activity of strobilurins is brought about by the β-methoxy-acrylate toxophore preventing the synthesis of adenosine triphosphate by targeting the Qo site of complex III of the mitochondrial electron transport chain [131].

The synthesis of dihydroxerulin was first described by Siegel and Brückner [132]. It began with stereoselective preparations of phosphorus ylide 1 and lactone aldehyde 2 and ended with a Wittig reaction between these entities. The process resulted in the formation of up to 30% of the trans Z isomer 3, along with up to 25% of a mixture of at least two stereoisomers. The same researchers synthesized xerulin via a convergent route and were able to get the pure form of the compound [132]. The total synthesis of xerulin by Negishi et al. [133] was accomplished from commercially available (E)-1-bromopropene, acetylene, and propynoic acid with 30% overall yield and >96% stereoselectivity.

## 7. Market and Commercialization

**Figure 10:**
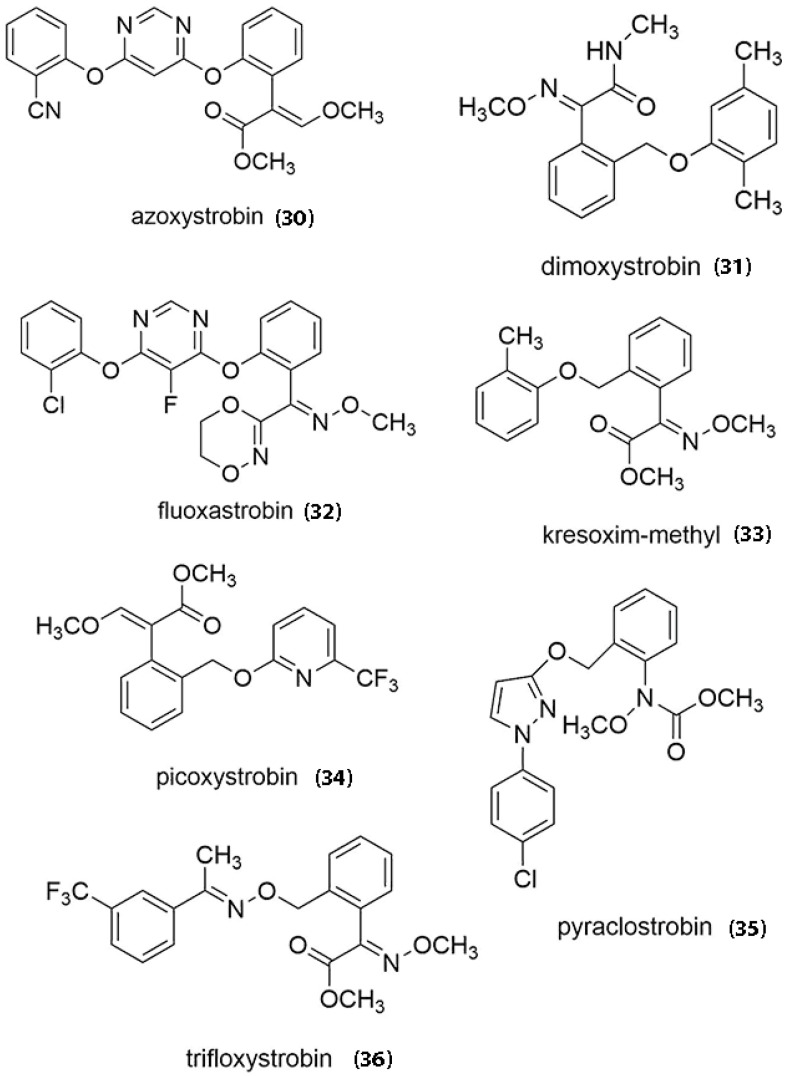
Chemical structures of synthetic strobilurins on the market.

Among the many compounds isolated from the OX genera, only strobilurins have made it to the commercial market, but the natural products were too unstable in the field experiments and it was considered a great challenge to achieve the biotechnological production of the natural compounds in ton scale as generally required for agrochemical fungicides. The discovery of the strobilurins, however, allowed the opportunity to develop synthetic fungicides by mimetic synthesis because the natural core structure was relatively simple [5]. The first synthetic fungicide arising from mimetic synthesis using strobilurins as a template was published in 1996 [134]. Natural strobilurins were named consecutively according to the order of discovery such as strobilurin A, B, and C. Applying Quantitative Structural Activity Relationship on the structures of the natural strobilurins, numerous companies were able to produce synthetic analogues, which are more effective in combating target organisms [103]. Nofiani et al. [84] stated that there are eight synthetic strobilurins on the market worldwide, some of which are already registered for agro-chemical use. The key compounds are azoxystrobin (30), di-moxystrobin (31), fluoxastrobin (32), kresoxim-methyl (33), pyraclostrobin (34), picoxystrobin (35) and tri-floxystrobin (36) [135] (Figure 10). The estimated worth of these synthetic compounds is $3.4 billion in 2015, making up to 25% of the fungicide market and 6.7% of the total crop protection market [84]. Currently, China is able to produce 4 strobilurin fungicides namely azoxystrobin, pyraclostrobin, tri-floxystrobin and kresoxim-methyl (Figure 10). Some synthetic strobilurins as fungicides with brand names, used against pumpkin diseases in Mississippi, were also listed [136]. Many of these synthetic fungicides have been developed from azoxystrobin. Azoxystrobin is a broad-spectrum fungicide with activity against several diseases on many edible crops and ornamental plants such as rice blast, rusts, downy mildew, powdery mildew, late blight, apple scab, and *Septoria* [137]. Amaro et al. [138] also showed that applying pyraclostrobin can enhance productivity and increase the antioxidative system, thereby reducing stress in Japanese cucumber (*Cucumis sativus*).

In general, these properties have made the strobilurins one of the most commercially successful natural product based class of agrochemicals and products from all the major agro-companies, which have distributed them for decades. However, the pathogenic fungi and oomycetes have increasingly developed resistance against the beta-methoxy-acrylates, and therefore it is advisable to use them in combination with other antifungal agents [5]. It is urgent to develop new antifungal pesticides in the near future based on different compound classes and with different modes of action, and fungi appear to be a very promising source for these. Therefore, the quest for antifungal metabolites from hitherto untapped sources should be intensified.

Other compounds from OX genera, though thoroughly studied in terms of bioactivities, have not yet been introduced to the market. Further research is needed in order to introduce these compounds for pharmaceutical, nutraceutical and medicinal applications.

## 8. Future Perspectives

Mushrooms have been shown to have profound health-promoting benefits and are commonly use in cosmetics [2,139]. The medical efficacy of bioactive compounds extracted from mushrooms is well-known. With advancements in chemical technology, it is now possible to isolate and identify relevant compounds such as polysaccharides, glycoproteins, and other bioactive compounds [140].

*Oudemansiella, Xerula, Mucidula* and *Hymenopellis*, among other genera of mushrooms produce important bioactive compounds, confirming their efficacy as antimicrobial, anti-oxidants and anti-inflammatory agents. The compounds isolated were mostly dominated by strobilurins; however, studies also identified xerulins, scalusamides, phenol A acid, di-hydro-citrinone and polysaccharides with bioactivities and have not been fully exploited. These promising compounds may have a bright future in pharmaceutical, nutraceutical and medicinal application. These future applications, however, face challenges. Studies on the cultivation of the OX genera are few as cultivation is generally developed only edible species. The OX complex are only cultivated in Asian countries. Therefore, thorough studies on the optimum cultivation methods of important species with bioactivities are necessary for stable bioactive sources to supply future demands. Mushrooms grown in greenhouses do not comply with current Good Manufacturing Practice (cGMP) requirements. Mushrooms should be grown in submerged cultures in a sterile environment to produce high quality bioactive compounds for pharmaceutical and medical applications. Another challenge is that the content of bioactive ingredients varies widely depending on the procedure, harvest, extraction time, and other environmental factors. Therefore, establishing a stable protocol considering important physical parameters is necessary. Furthermore, bioactivities of mushrooms are usually demonstrated using crude extracts, with a mixture of solvents and other metabolites.

Some compounds, especially strobilurins, have already been identified with structure elucidation but many are strongly cytotoxic. However, it is possible that other interesting compounds may be isolated from OX taxa in the future. Polysaccharides, for instance, are non-toxic and should be further explored for their bioactivities. The possible side effects of these compounds are an important concern and must be studied in order to upgrade for pharmaceutical applications. It is also essential to establish and validate standard testing protocols to guarantee the quality of the bioactive compounds isolated for pharmaceutical applications, thus further studies are necessary.

## Figures and Tables

**Table 1:** History of taxonomic placements of oudemansielloid/xeruloid (OX) genera.

Author	Year	Arrangement	Adopted
Spegazzini [16]	1880	Initially proposed *Oudemansia* to accommodate a single species, *Agaricus platensis* Speg.	
Spegazzini [17]	1881	Changed the name to *Oudemansiella*	
Patouillard [42]	1887	Erected *Mucidula* to separate *Agaricus mucidus* from both *Collybia* (Fr.) Kummer and *Armillaria* (Fr.) Kummer based on the presence of velar layers and the voluminous spores	
Hoehnel [43]	1910	Emended *Oudemansiella* to include species with velar layers, a gelatinized pileipellis, and large cystidia and spores	
Boursier [44]	1924 1924	Expanded *Mucidula* to include *Collybia radicata* (Relhan: Fr.) Quel. and *C. longipes* (Bull.) Kummer, emphasizing morphological similarities (spores, basidia, cystidia, hymenioderm)	
Maire [45]	1933	Separated *C. longipes* from *Mucidula* and proposed the new genus *Xerula*	Singer [46,47]
Moser [18]	1955	Merged *Xerula* and *Mucidula* into *Oudemansiella*	Singer [19,20,21]
Clémençon [22]	1979	Treated *Xerula* as one of the 5 subgenera of *Oudemansiella*	
Dörfelt [23]	1980	Retained *Oudemansiella* and *Xerula* as independent genera	Boekhout & Bas [24], Redhead et al. [25], Petersen & Halling [29], Petersen & Methven [30], Corner [32], Contu [33], Mueller et al. [34], Petersen & Nagasawa [26], Petersen & Baroni [27], Petersen [28,31]
Pegler & Young [35]	1987	Divided *Oudemansiella* into five sections under the subgenera *Oudemansiella* and *Xerula*	Rexer & Kost [36,37], Yang & Zang [38], Yang [39], Mizuta [40]
Yang et al. [41]	2009	Divided *Oudemansiella* into four sections (*Oudemansiella, Mucidula, Dactylosporina* and *Radicatae*)	
Petersen & Hughes [10]	2010	Introduction of four new genera (*Hymenopellis, Paraxerula, Ponticulomyces, Protoxerula*) and reconfiguration of other genera such *Dactylosporina, Mucidula, Oudemansiella* and *Xerula*	accepted until now

**Table 2:** Some cultivable OX species on different substrates and (%) biological efficiency, as effectiveness of mushroom strain growth in the given substrate. Biological efficiency refers to the percentage of ratio of fresh mushroom weight over the dry weight of the respective substrate.

Species	Substrate	Biological Efficiency (%)	References
*Hymenopellis radicata*	Oak sawdust	–	Shim et al. [69]
	Sawdust	100	Gao [70]
*Mucidula mucida*	Oak sawdust	–	Lee et al. [71]
*Oudemansiella canarii*	Sugar-cane bagasse	55.66	Silveira Ruegger et al. [72]
	Eucalyptus sawdust	19.51	
	Cottonseed hull	113.64	Xu et al. [73]
	Corncob	105.65	
	Sawdust	85.49	

**Table 3:** Secondary metabolites produced by some species of the OX complex with their bioactivities.

Species	Bioactive Compounds	Biological Activities	References
*Mucidula mucida*	Mucidin/strobilurin A/mucidermin	Antifungal	Musilek et al. [77], Anke et al. [98], Subik et al. [104]
	Strobilurins	Antifungal	Iqbal et al. [99], Anke et al. [98]
	Oudemansins	Cytotoxic	Ying et al. [105]
		Antifungal	Vondracek [106], Anke et al. [100]
	Strobilurin X, 4’-methoxymucidin		Anke et al. [107]
*Hymenopellis radicata*	Oudemansin X		Anke et al. [107] Umezawa et al. [90], Tsantrizos et al. [90,107]
	Strobilurins	Antifungal	
	Oudemansins	Antihypertensive	
	Oudenone	Hemagglutinating activity	Liu et al. [92]
	Lectin	Antifungal	Anke et al. [100,104,108]
	Mucidin	Antioxidative, anti-inflammatory, lung-protective effects	Gao et al. [91]
	SMPS, MPS (mycelia polysaccharides)	Antioxidant; antifungal	Wang et al. [9], Zou [109], Liu et al. [110]
	Polysaccharides	Antifungal	Rosa et al. [14,96]
*Oudemansiella canarii*	Oudemansin A	Antifungal, inhibitor of eucaryotic respiration	Anke et al. [108]
*Oudemansiella melanotricha*	Oudemansin B, strobilurin C	Inhibitor of cholesterol biosynthesis	Kuhnt & Anke [111]
	Xerulin, di-hydro-xerulin, xerulinic acid	Antifungal	Weber et al. [112]
	Hydroxy-strobilurin D	Antifungal, inhibitor of eucaryotic respiration	Anke et al. [108]
*Xerula longipes*	Oudemansin B, strobilurin C	Antifungal	Sivanandhan et al. [88]
*Xerula pudens*	Strobilurin C	Antifungal	Sivanandhan et al. [88] Sadorn et al. [113]
	Oudemansin B	Antimalarial, antifungal, cytotoxic	
*Xerula* sp. BCC56836	Oudemansins	Antibacterial	Sadorn et al. [113]
	Strobilurin derivatives	Antibacterial, antifungal	
	Scalusamides A	Enzyme-inhibitory activity; antifouling activity	
	Phenol A acids	Enzyme-inhibitory activity	
	Dihydro-citrinone	Antibacterial	
	Xerulins	Antibacterial	
	Xerucitrinic acid A	Antimicrobial, insecticidal	
	2-(5-Heptenyl)-6,7,8,8a-tetrahydro-3-methyl-4H-pyrrolo [2,1-b][1,3]oxazin-4-one (17)		
